# The temporal dynamics of Large‐Scale brain network changes in disorders of consciousness: A Microstate‐Based study

**DOI:** 10.1111/cns.14003

**Published:** 2022-11-01

**Authors:** Chunyun Zhang, Yi Yang, Shuai Han, Long Xu, Xueling Chen, Xiaoli Geng, Li Bie, Jianghong He

**Affiliations:** ^1^ Department of Neurosurgery The First Hospital of Jilin University Changchun China; ^2^ Department of Neurosurgery Beijing Tiantan Hospital, Capital Medical University Beijing China; ^3^ Chinese Institute for Brain Research Beijing China; ^4^ Beijing Institute of Brain Disorders Beijing China; ^5^ China National Clinical Research Center for Neurological Diseases Beijing China

**Keywords:** disorders of consciousness, microstate, temporal dynamics

## Abstract

**Background and Objectives:**

The resting‐state brain is composed of several discrete networks, which remain stable for 10–100 ms. These functional microstates are considered the building blocks of spontaneous consciousness. Electroencephalography (EEG) microstate analysis may provide insight into the altered brain dynamics underlying consciousness recovery in patients with disorders of consciousness (DOC). We aimed to analyze microstates in the resting‐state EEG source space in patients with DOC, the relationship between state‐specific features and consciousness levels, and the corresponding patterns of microstates and functional networks.

**Methods:**

We obtained resting‐state EEG data from 84 patients with DOC (27 in a minimally conscious state [MCS] and 57 in a vegetative state [VS] or with unresponsive wakefulness syndrome). We conducted a microstate analysis of the resting‐state (EEG) source space and developed a state‐transition analysis protocol for patients with DOC.

**Results:**

We identified seven microstates with distinct spatial distributions of cortical activation. Multivariate pattern analyses revealed that different functional connectivity patterns were associated with source‐level microstates. There were significant differences in the microstate properties, including spatial activation patterns, temporal dynamics, state shifts, and connectivity construction, between the MCS and VS groups.

**Discussion:**

Our findings suggest that consciousness depends on complex dynamics within the brain and may originate from the anterior cortex.

## Graphical abstract

Consciousness is dependent on complex dynamics within the brain and that it may originate from the anterior cortex. The feathers of the source‐level microstate may be generalizable markers for consciousness during the generation and recovery processes.

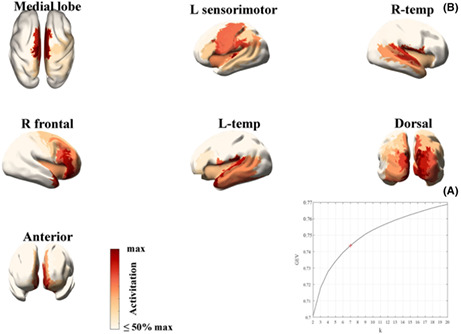

## 1 INTRODUCTION

Disorders of consciousness (DOC) are caused by direct disturbances in neural systems that regulate arousal and awareness or indirectly by disruptions in their connections.^1^ DOC include coma (unwakefulness and reflex behaviors only), unresponsive wakefulness syndrome (UWS; previously known as vegetative state [VS]; wakefulness but reflex behaviors only), and minimally conscious state (MCS; clinical presentation of signs of consciousness). Once patients with DOC recover functional communication or object use, they are considered emerging from a minimally conscious state (eMCS). The Consciousness Recovery Scale‐Revised (CRS‐R) is a reliable and sensitive clinical tool that can discriminate among patients with UWS, MCS, and eMCS.^2^, ^3^, ^4^, ^5^ Although behavioral assessment remains the gold standard, neuroimaging techniques provide objective evidence of CNS damage after brain injury.^1^, ^3^, ^4^

Brain networks in patients with DOC are mostly examined based on functional connectivity and spectral energy; however, temporal dynamics and spatiotemporal interaction effects in these patients remain unclear.^4^, ^5^, ^6^, ^7^, ^8^ These can be examined through electroencephalography (EEG) microstate analysis, which is used to study brain states and their fast transitions in cognition and disease. Since EEG can record fluctuations within a millisecond timescale, it is suitable for investigating brain resting states and their effects on responses to stimuli.^9^, ^10^ On EEG, only a few topographic classes remain stable for 10–100 ms before rapidly shifting to other classes. Microstates have been termed “atoms of thought” since they are considered electrophysiological correlates of brain activity that is rapidly activated and inactivated during the resting state.^11^ There are microstate alterations during healthy development and aging and in various neurological disorders, including schizophrenia, depression, and frontotemporal dementia.^12^, ^13^, ^14^ Therefore, EEG microstates can facilitate the investigation of changes in brain dynamics underlying impaired information processing in patients with DOC on a millisecond timescale. Additionally, EEG microstates are suitable candidate biomarkers with a faster temporal scale than power spectral or functional network analysis.^15^

The use of microstates allows a computationally optimized method for capturing cortical activity from an entire EEG montage in studies on disease abnormalities. The four canonical prototypes commonly appear in awake EEG; however, microstates can be clustered from EEG recordings without a priori assumptions regarding the number of prototypical maps. Instead, goodness‐of‐fit measures are used to determine the optimal number of topographies for each study. This data‐driven approach is helpful for studies on brain disorders exhibiting clinical symptoms due to aberrant networks.^16^

Dynamic functional connectivity is a recurrent state of brain connectivity measurable on resting‐state fMRI. It is associated with different cognitive and pathological states. Moreover, it is a potential disease biomarker; however, the underlying neuronal mechanisms remain unclear.^17^ Microstate analysis is typically performed at the sensor level, thereby providing limited anatomical insight. There is a need to elucidate the mechanisms underlying discrete brain‐state generation, which could improve the current understanding of behavior, cognition, and neurological diseases as well as DOC. Several studies have combined sensor‐space microstate analysis with source reconstruction to reconstruct the electrophysiological sources underpinning microstate maps. However, this approach could limit insight into functional brain states for several reasons. First, given that the inverse problem is not unique, multiple active networks may be related to each sensor‐level microstate because different spatial brain activation patterns may generate similar topographical maps. Second, as the spatial blurring in EEG resulting from volume conduction through tissues has different conductivity levels, EEG topographies have limited spatial resolution and may not distinguish finer differences across maps. Indeed, EEG topographies can be sufficiently obtained using only eight electrodes, which suggests that the topographies do not contain much spatial information. Finally, the sensor‐space EEG microstate maps underestimate the importance of non‐occipital or non‐alpha‐band networks given the dominance of alpha‐band occipital sources in the sensor‐space eyes‐closed resting‐state EEG, which could be attributed to the head shape and forward model.^18^

This study aimed to analyze microstates in the resting‐state EEG source space in patients with DOC to validate differences in the relevant consciousness levels. We aimed to investigate the relationship between state‐specific features and consciousness levels and explore the corresponding patterns of microstates and functional networks. Our findings could provide insight into the relationship between spontaneous microstates and human consciousness.

## 2 MATERIALS AND METHODS

### 2.1 Patients and diagnosis

We included 96 patients with DOC from the Department of Neurosurgery of the Beijing Tiantan Hospital, Capital Medical University. The patients were diagnosed with VS/UWS or MCS based on the CRS‐R with an awakening stimulation protocol, if necessary. The clinical diagnosis was established based on the consciousness level. The CRS‐R was readministered four times during the week after the EEG recording to validate the consistency of the clinical diagnosis.^19^

The inclusion criteria were as follows: (1) no scalp lesions or intracranial metal implants, (2) no history of neurological or psychiatric disorders, (3) no acute illness or episodes of chronic illness, and (4) DOC lasting >28 days.

The exclusion criteria were as follows: (1) the presence of intracranial anterior and posterior lobe lesions; (2) a history of having a pacemaker, aneurysm clips, or other metallic devices; and (3) incomplete cranial bone.

Informed consent was obtained from all participants before the study commencement and they were free to withdraw at any time. All procedures were conducted following the relevant guidelines and regulations as well as the Declaration of Helsinki. This study was approved by the local ethics committees of the Beijing Tiantan Hospital, Capital Medical University.

### 2.2 EEG recording and processing

We recorded 64 channels of EEG data at a sampling rate of 2500 Hz using a 64‐channel EEG recorder (BrainAmp 64 MRplus, BrainProducts) equipped with sintered Ag/AgCl pin electrodes. Each recording lasted for 10 min. In some patients, the awakening stimulation protocol for the CRS‐R assessment was applied immediately before EEG acquisition, with the patient remaining awake. In case of prolonged eye closure or sleep features (including sleep spindles or K complex waves in the EEG), the EEG recording was terminated and the data was discarded. Subsequently, the CRS‐R awakening protocol (with EEG acquisition) was repeated. Offline preprocessing was performed using Fieldtrip running in a MATLAB environment (MathWorks Inc., Natick, MA, USA) with self‐programmed scripts. The data were bandpass filtered at 1–100 Hz, notch filtered at 50 Hz to remove line noise, and down‐sampled to 250 Hz. Independent component analysis decomposition was used to remove visual and cardiac artifacts. Each scan removed between two and six components from the resting‐state data. Data with <80% retention were considered invalid and not included in subsequent analyses.

### 2.3 MR recording and source‐space microstate pipeline

#### 2.3.1 MR recording

A three‐dimensional gradient echo T1 anatomical scan (TR, 1800 ms; TE, 2.5 ms; thickness, 1.0 mm; spacing, 1.0 mm; slices, 176; duration, 4 min and 18 s) was obtained for source reconstruction.

#### 2.3.2 Source reconstruction and microstate creation

Optimal microstate numbers were determined using all the scans for backfitting. MRI preprocessing was performed using the Fieldtrip toolbox (Version 20,191,014) running in a MATLAB environment (MathWorks Inc.). Source reconstruction was performed using the eLORETA algorithm implemented in FieldTrip. eLORETA was chosen based on a systematic evaluation of source reconstruction of resting‐state EEG, where it demonstrated high performance in a range of metrics, especially with parcellated data, which is similar to our present study.^20^ The entire T1 volume was resliced onto a 1 × 1 × 1 mm^3^ grid aligned with the coordinate axes. From the T1‐weighted MRI images, we extracted the scalp, brain, and cortical surfaces using FreeSurfer. Vertices of the cortical surface were labeled based on the AAL78 atlas in the +microstate toolbox by taking the time course of the first principal component of all voxels within a region of interest.^21^ The 78 region AAL atlas was the same as in Fieldtrip just excluding subcortical regions such as the thalamus and hippocampus. Source time courses were bandpass‐filtered in the 1–30 Hz frequency band.^18^ For each scan, we randomly selected 5000 global field powers (GFP) to determine the optimal number of microstates. The microstate maps were identified from 420,000 GFP peaks; moreover, microstate labels were assigned to each sample of the full scan. Each GFP peak in the full dataset was labeled as a state based on the microstate centroid map with the minimum distance. All other samples were assigned the same state label as that of the nearest GFP peak.^18^ The kneedle algorithm showed that seven states were optimum; therefore, we proceeded to back‐fit the results of the seven‐state clustering to the full EEG scans.^22^

#### 2.3.3 Microstate statistics

Global statistics of the microstate sequences included the global explanation variance (GEV), mean duration of microstates, and Hurst exponent of the sequences. Class‐specific statistics included the mean duration of the microstates within a particular class, class coverage (percentage of time spent within a class), and class occurrences (frequency of state appearance [per second]). Furthermore, we calculated the non‐random microstate syntax.^23^

### 2.4 MVPA testing for functional connectivity in each microstate

After filtering the data into four narrow bands (delta 1–4 Hz, theta 4–8 Hz, alpha 8–13 Hz, and beta 13–30 Hz), the Hilbert transform was calculated. For a given microstate, all analytic signals within the microstate class were concatenated. Subsequently, the samples were epoched into non‐overlapping 5‐s windows, and phase synchronization was calculated using the weighted phase lag index (wPLI). This method was repeated for each microstate class to obtain functional connectivity patterns.

To test the hypothesis that microstates are significantly associated with functional connectivity, we applied a multivariate pattern analysis (MVPA) using the “networks_wpli” algorithm in the +microstate toolbox. Weighted degree distributions were used as features in a multiclass classifier for the microstate labels, and the results were five‐fold cross‐validated. A permutation test was used to test the significance of classification accuracy.^23^

### 2.5 Statistical analyses

Statistical analyses of behavioral data and microstate indicators were performed using R statistical software (The R Project for Statistical Computing, Vienna, Austria). Shapiro–Wilk tests were performed to analyze distributional characteristics. The Wilcoxon rank‐sum test was used to examine microstate features compared with baseline microstate indicators and demographic characteristics. The false discovery rate (FDR) correction was used to control for the multiple comparison problem across all seven tests among the three microstate indicators. The microstate syntax changes between microstates in different levels of consciousness. The p‐values of state shifts between states were FDR‐corrected.

## 3 RESULTS

### 3.1 Epidemiology characters

Between October 2018 and September 2021, among 96 eligible patients with DOC, we included a final cohort of 84 patients with DOC (MCS, *n* = 27; VS, *n* = 57). We excluded 12 patients due to incomplete skulls, lack of individual MRI data, and artifacts in the EEG signals. Regarding sex, there were 16 and 34 women with MCS and VS, respectively (*p* = 1.000). The mean age of the MCS and VS groups was 44 ± 14.2 and 45.6 ± 14.9 years, respectively (*p* = 0.627). Regarding etiology, the MCS group had 8, 12, and 7 cases of hypoxia, stroke, and trauma, respectively, while the corresponding values in the VS group were 18, 27, and 12, respectively (*p* = 0.883). The mean post‐injury duration in the MCS and VS groups was 15.9 ± 36.3 and 6.03 ± 7.75 months, respectively (*p* = 0.171). There were no significant between‐group differences in the baseline clinical characteristics. Table 1 shows the epidemiological characteristics of the enrolled patients.

**TABLE 1:** Summary of patients' characteristics by groups

	ALL	MCS	VS	*p*
	*N* = 84	*N* = 27	*N* = 57	
Gender				1.000
Female*	50 (59.5%)	16 (59.3%)	34 (59.6%)	
Male*	34 (40.5%)	11 (40.7%)	23 (40.4%)	
Age (year)^十^	45.1 (14.6)	44.0 (14.2)	45.6 (14.9)	0.627
Etiology				0.883
Anoxia*	26 (31.0%)	8 (29.6%)	18 (31.6%)	
Stroke*	39 (46.4%)	12 (44.4%)	27 (47.4%)	
TBI*	19 (22.6%)	7 (25.9%)	12 (21.1%)	
Post‐injury(m)^十^	9.21 (21.8)	15.9 (36.3)	6.03 (7.75)	0.171

Abbreviations: MCS, minimal consciousness state; VS, vegetative state.

*n (%), ^十^mean (standard deviation).

### 3.2 Resting‐state microstate maps

**FIGURE 1:**
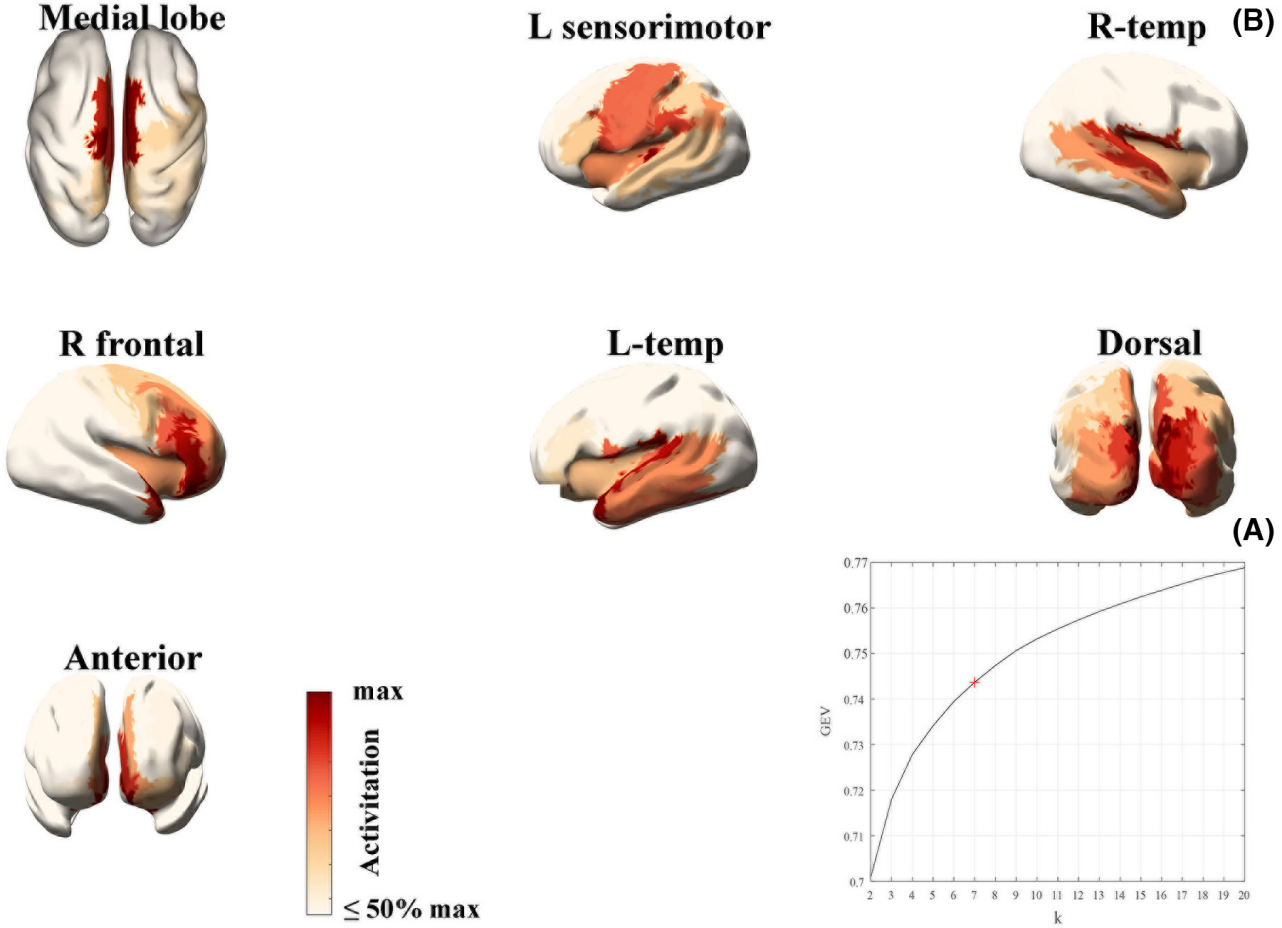
Resting‐state microstates. (A) GEV vs. number of states (k) for resting‐state data. Here, GEV is calculated across the 420,000 peaks used for clustering. The kneedle algorithm found that k = 7 states was optimum, marked by a red “+”. (B) Resting‐state microstate maps derived from the k‐means clustering algorithm for k = 7 states

We source‐reconstructed the EEG resting‐state data from the 84 eligible patients. Based on a sample of 420,000 GFP peaks, microstates were calculated using the *k*‐means clustering algorithm for all patients. Figure 1A shows the GEV across these 420,000 peaks as 𝑘 varied from 2 to 20 states. Regarding the MCS scans from which the GFP peaks were sampled to estimate microstates, seven states had a GEV of 70.7 ± 1.13%. There was no significant change in the GEV in the VS scans (71.1 ± 1.52%, 𝑝 = 0.4322, Wilcoxon sign‐rank test). Figure 1B shows the spatial map of each empirical EEG source‐level microstate. Seven maps were identified, including the medial lobe state, left sensorimotor state, right temporal state, right frontal state, left temporal state, dorsal state, and anterior state. Figure S1 shows the differences in the duration, occurrence, and coverage of the seven microstates across all patients.

### 3.3 Statistics of resting‐state microstate sequences

**FIGURE 2:**
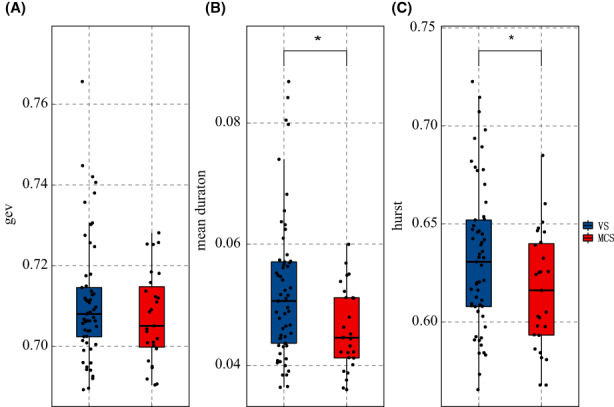
CEV, mean duration, and Hurst exponent of each group (VS MCS). (A) There is no significant change in GEV between two groups (*p* = 0.432). (B) MCS has significantly decreased mean duration (*p* < 0.001) compared with VS. (C) MCS has significantly decreased hurst exponent (*p* = 0.022) compared with VS. *p < 0.05 **p < 0.01 ***p < 0.001

**FIGURE 3:**
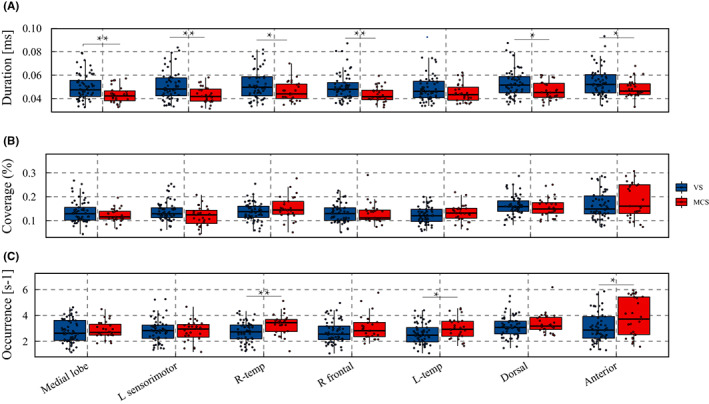
Duration, coverage, and occurrence of each of the seven resting‐state microstate maps in two groups. (A) MCS has significantly decreased duration in medial lobe state (*p* = 0.008 FDR‐corrected), L sensorimotor state (*p* = 0.008 FDR‐corrected), R‐temp state (*p* = 0.035 FDR‐corrected), R frontal state (*p* = 0.008 FDR‐corrected), dorsal state (*p* = 0.012 FDR‐corrected), and anterior state (*p* = 0.028 FDR‐corrected) compared with VS. For the duration of L‐temp state, MCS was nearly significant (*p* = 0.051 FDR‐corrected). (B) No significant difference was observed between the two groups in state coverage. (C) MCS had significantly increased occurrence in R‐temp sate (*p* = 0.008), L‐temp state (*p* = 0.044), and anterior state (*p* = 0.049). *p < 0.05 FDR‐corrected, **p < 0.01 FDR‐corrected

We evaluated microstate changes in each group. Figure 2 shows that the MCS group had a significantly decreased mean duration (*p* < 0.001) and Hurst exponent (*p* = 0.022) compared with the VS group. As shown in Figure 3A, compared with the VS group, the MCS group had a significantly decreased duration in the medial lobe state (*p* = 0.008 FDR‐corrected), L sensorimotor state (*p* = 0.008 FDR‐corrected), R‐temp state (*p* = 0.035 FDR‐corrected), R frontal state (*p* = 0.008 FDR‐corrected), dorsal state (*p* = 0.012 FDR‐corrected), and anterior state (*p* = 0.028 FDR‐corrected). As shown in Figure 3B, the MCS group had a significantly lower coverage in the L‐sensorimotor state (*p* = 0.034 uncorrected) than the VS group. As shown in Figure 3C, the MCS group had a significantly increased occurrence in the R‐temp state (*p* = 0.008 FDR‐corrected), L‐temp state (*p* = 0.044 FDR‐corrected), and anterior state (*p* = 0.049 FDR‐corrected) compared with the VS group.

**FIGURE 4:**
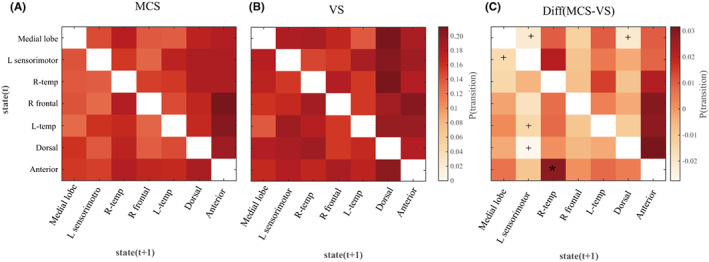
Syntax of resting‐state microstates. (A) The average syntax matrix of MCS. (B) The average syntax matrix of VS. (C) The difference in the syntax matrix of MCS‐VS. MCS has a significantly lower transition probability from the L sensorimotor state to the anterior state; medial lobe, L‐temp state, and dorsal state to L sensorimotor sate; and medial lobe state to dorsal state with no multiple comparison correction. MCS had a significantly higher transition probability from the anterior state to the R‐temp state. * *p* < 0.05 FDR‐corrected, + *p* < 0.05 uncorrected

Among the seven microstates, the dorsal state had the highest coverage and the highest number of occurrences per s in the VS group (Figure 3). This can be observed in the group‐level syntax matrices (Figure 4A), in which almost all states tended to predominantly transition to the dorsal state. The anterior state had the highest coverage and highest number of occurrences per second in the MCS group (Figure 3). This can be observed in the group‐level syntax matrices (Figure 4B), in which almost all states tended to predominantly transition to the anterior state. Regarding between‐group comparisons of the syntax matrix, Figure 4C shows that compared with the VS group, the MCS group had a lower transition probability from the L sensorimotor state to the anterior state (*p* = 0.034 uncorrected); from the medial lobe state, L‐temp state, and dorsal state to the L sensorimotor state (*p* = 0.013 uncorrected, 0.020 uncorrected, and 0.022 uncorrected, respectively); and from the medial lobe state to the dorsal state (*p* = 0.049 uncorrected). Compared with the VS group, the MCS group had a significantly higher transition probability from the anterior state to the R‐temp state (*p* = 0.048 FDR‐corrected).

### 3.4 Microstate‐specific functional connectivity

**FIGURE 5:**
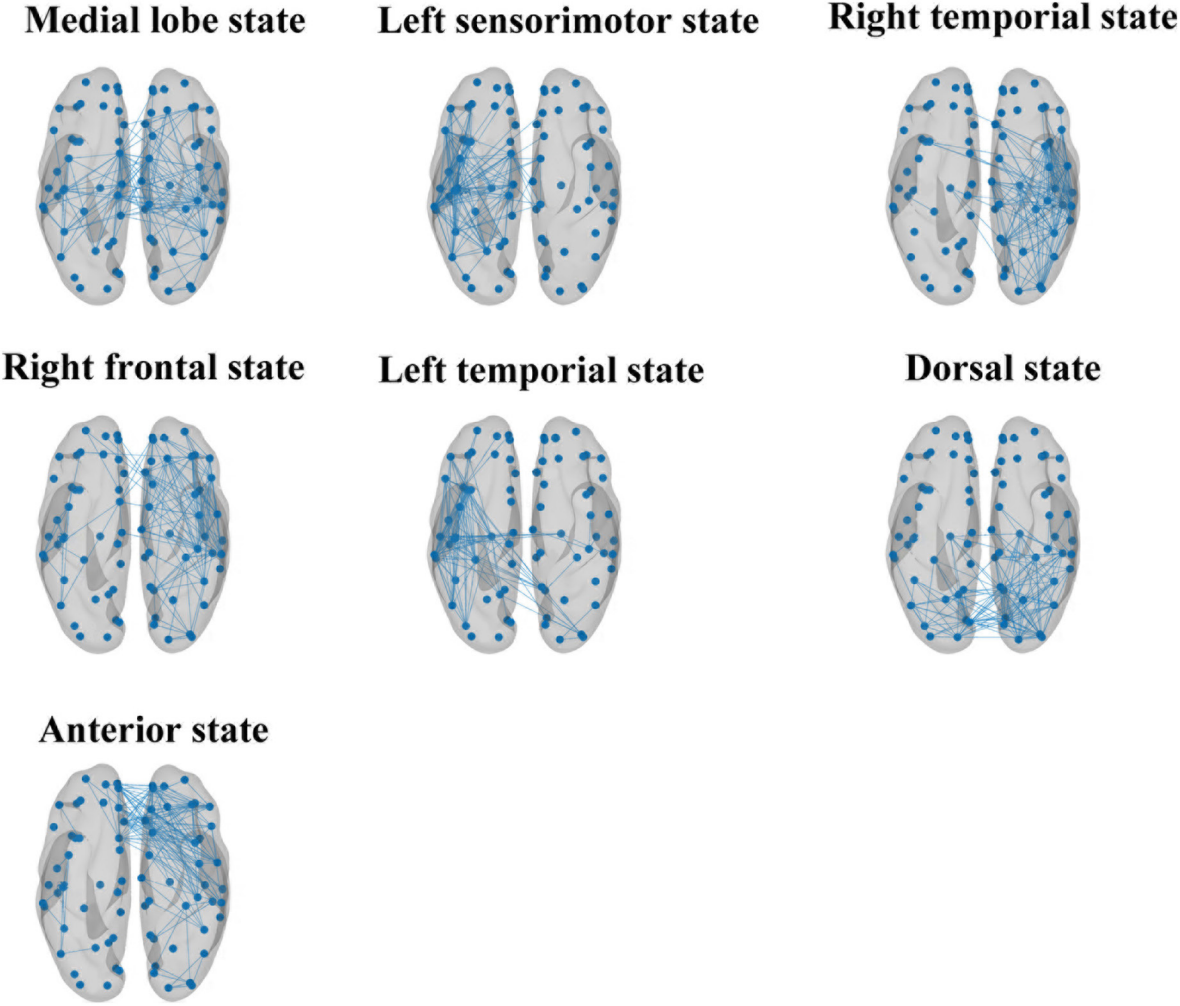
Functional connectivity patterns. Edges show the top 1% of edges that deviate from static background connectivity

Finally, we tested the hypothesis that different microstate classes are associated with distinct functional connectivity patterns and reflect rapid transitions in dynamic phase synchronization patterns in the brain. We used multiclass MVPA to test whether microstates could be predicted using the microstate‐segmented wPLI connectivity matrices. Table 2 presents the classification accuracy and permutation testing p‐values for each frequency band. The classification accuracy was significantly above the chance level of 1/7 (*p* < 0.001, 1000 permutation tests from 1000 surrogates), which suggests distinct functional connectivity patterns among the microstates. Figure 5 shows the structure of the alpha‐band network.

**TABLE 2:** MVPA classification statistics for microstate‐segmented connectivity

Band	Accuracy	*p*‐value
Delta	0.2250	<0.001
Theta	0.1799	<0.001
Alpha	0.1770	<0.001
Beta	0.1900	<0.001

## 4 DISCUSSION

Several studies have investigated microstates in patients with DOC at the sensor level in EEG. Microstates show specific temporal dynamics, spatial activation, and correlations with different types of information processing.^17^ We detected seven microstates in the resting‐state brain, including the medial lobe, left sensorimotor, right temporal, right frontal, left temporal, dorsal, and anterior states, using source‐level EEG. The seven key states identified specific spatial activation patterns and temporal features in the EEG source space. We analyzed the microstates with respect to duration, coverage, occurrence, and syntax. There were significant between‐group differences (MCS vs. VS/UWS) in the features of the transient states, which suggests a physiologically relevant relationship between microstates and human consciousness. The MVPA results suggested that each microstate corresponds to a specific brain network. There was no significant between‐group difference in the GEV, which suggests that the seven microstates could explain the EEG time series in both groups to approximately the same extent. The MCS group showed a shorter mean duration of the seven microstates than the VS group, which suggests that within the same network mode or microstate, information transmission and processing in patients with VS may take longer than in patients with MCS. For each state, except for the L‐temp state, all other states in the MCS group had a shorter duration. This may correspond to a higher incidence of some states, such as the R‐temp, L‐temp, and anterior states. Peng Gui et al. performed microstate analysis at the sensor level in patients with DOC. The results were similar to our results, which showed that the lower the level of consciousness, the longer the mean duration of the microstate.^24^ Lucie Bréchet et al. also found that the mean duration of all microstates in the sensor level was longer under sleep and deep anesthesia.^25^ The above studies further confirm that longer mean durations of microstates may represent lower brain dynamics in consciousness‐related studies.

The human resting‐state network mainly comprises multiple distinct areas of association in the cerebral cortex. The anterior forebrain mesocircuit and frontoparietal network are consistently implicated in the restoration of cerebral activity during recovery from DOC. Corticothalamic projections parallel the striatopallidal negative feedback loop, which influences thalamic outflow back to the cortex and striatum from the anterior forebrain circuit.^26^, ^27^ There are two subnetworks within the frontoparietal network, namely, the default mode network (DMN) and the executive control network (ECN). The DMN mediates inner awareness and self‐related processes through the midline nodes located in the medial prefrontal cortex, posterior cingulate, and precuneus. Moreover, the ECN, which is located in the dorsolateral prefrontal and posterior parietal cortices, mediates attention and awareness of the environment.^28^ Taken together, the activated regions of the medial lobe and right frontal states involved the saline network; the left sensorimotor state involved the ECN; the right and left temporal states involved the DMN; the posterior state involved the visual network as well as the posterior cingulate gyrus and precuneus of the DMN; and the anterior state involved the medial prefrontal lobe of the DMN. Preservation of DMN function facilitates consciousness recovery following acute DOC. Additionally, DMN activity is closely linked to the consciousness‐level awareness of patients, which is considered an important indicator for distinguishing MCS from VS/UWS.^29^, ^30^, ^31^, ^32^, ^33^ The MCS group had a higher occurrence of the R‐temp, L‐temp, and anterior states than the VS group. The activated regions of the aforementioned belonged to the DMN. This further demonstrates that consciousness recovery may be related to the retention of the DMN function.

Therefore, it is important to determine whether any particular rules exist for the temporal structure of such alternations. Transitions between stable states may rapidly occur between segments of quasi‐stable topographies within the microstates. This description of the temporal dynamics of brain processes is consistent with several theoretical models that suggest that conscious cognition is temporally discrete and can be divided into stable intervals termed “perceptual frames.” This concept has been further supported by evidence from numerous electrophysiological and imaging studies.^34^, ^35^ It is important to examine whether the sequence of microstates (“microstate syntax”) determines the consciousness level and is correlated with consciousness recovery. The dynamics of EEG microstate sequences can be described using scale‐free monofractal dynamics over a range of dynamic scales (from milliseconds to several seconds). Previous findings indicate that EEG microstate time series are perfectly self‐similar, i.e., they exhibit the same information when observed at various timescales.^36^, ^37^ Accordingly, the microstate time series present a structured temporal organization that is neither random nor predetermined but cannot be predicted. The scale‐free dynamics only merge when a system reaches a critical point, which indicates that the brain operates under conditions far from resting‐state homeostasis to maintain a high degree of responsiveness and flexible management of continuous information flow from multiple sources. Notably, the long‐range dependency of microstate sequences is crucially dependent on variability in individual microstate durations rather than the microstate sequence itself.^9^ In our study, the Hurst exponent for all patients was >0.5, which is suggestive of long‐range temporal correlations. Microstate duration is the most critical parameter; without this parameter, there is no remote dependency. This is in line with the notion that precise timing is essential to manage the continuous flow of information that the brain must process at all times to achieve perception, cognition, and ultimately consciousness.^37^ The difference in the mean duration of microstates between the two groups may have caused the difference in Hurst's index. Global statistics further confirmed the non‐stationary nature of resting‐state EEG. The significant between‐group difference in the Hurst exponent could be directly attributed to the microstate duration being shorter in the MCS group than in the VS group. Moreover, a shorter microstate duration facilitates the response to external information flow and may represent a higher consciousness level.

The microstate syntax matrix showed that the MCS group mainly showed a transition to the anterior state; in contrast, the VS group showed a higher probability of transition to the dorsal state and an approximately equal probability of transition to other microstates. The dorsal and anterior states involved the posterior cingulate/cuneus, anterior cingulate, and medial prefrontal lobes located in the DMN. Our research is the first example of source‐space EEG microstates and is an exploratory study. Although the MCS group had a non‐significantly higher probability of transition from the R‐temp, R‐frontal, L‐temp, and dorsal states to the anterior state than the VS group, this may represent partial evidence of the role of the medial prefrontal lobe in consciousness recovery. Compared with the VS group, the MCS group showed a significantly higher transition probability from the anterior state to the R‐temp state, which both belong to the DMN and anterior cortex. This suggests that the recovery of connections and transitions within the DMN may contribute to consciousness recovery and further emphasizes the importance of the anterior cortex. Although some of the results do not pass the FDR correction, we believed that the results still reflect some possible trends as an exploratory study. The MCS group had a lower probability of transition between the medial lobe state and the L sensorimotor state than the VS group. The VS group had an increased probability of transition from the L‐temp and dorsal states to the L sensorimotor state, which suggests that microstates in the VS group were more inclined to transition to the ECN rather than the DMN. In contrast, the MCS group showed a reduced probability of transition from the medial lobe state to the dorsal state, which suggests that the anterior cortex may be crucially involved in consciousness recovery within the DMN. Higher‐order theories (HOTs) suggest that consciousness depends on representations of lower‐order mental states. According to the HOT, the anterior cortex, especially the prefrontal cortex, could be crucially involved in higher‐order representations; however, this remains unclear.^38^, ^39^ Contrastingly, global workspace theories (GWTs) propose dividing the brain into specialized modules that perform different functions, with long‐distance between‐module connections; moreover, the frontoparietal region is considered the central point.^40^, ^41^ Our findings from the microstate transition matrix provide lateral evidence that consciousness may originate from the anterior cortex, which is consistent with both HOT and GWT. An important observation during consciousness is the anti‐correlation among the activities of different brain regions, which is consistent with the assumption by GWT that different information flow pathways in the brain have competing relationships in large network regions. The fMRI blood oxygen level‐dependent signal may manifest as mutual activity suppression in different cortical regions, which leads to anti‐correlated dynamics. Contrastingly, for microstates, this was manifested in the MCS group, which showed a higher and suppressed tendency of transition to the DMN and ECN, respectively. Thus, MCS had a higher awareness level than the VS group.^42^

In DOC, the brain network is characterized by restriction to the posterior cortex (with an inability to shift to the anterior cortex), loss of dynamics (reduced state transitions), and reduced information interactions (reduced connectivity with other brain regions). Accordingly, the hypodynamic and disconnected network is inconsistent with the state of human consciousness, which may be a neurophysiological mechanism of DOC.

Clustering in the source space, rather than in the sensor space, may have contributed to the increased number of states in our study. Forward projections of source dynamics to the sensor space exhibit a non‐uniform signal‐to‐noise ratio (SNR). Accordingly, states coming from low‐SNR cortex areas may be underrepresented in the sensor space maps. Furthermore, multiple source topographies may result in non‐unique maps due to the finite spatial sampling of EEG sensors. Therefore, multiple source‐space states may appear similar at the sensor level. Finally, the heterogeneity of brain injury areas in patients with DOC may contribute to this phenomenon.

All previous publications on source‐space microstates have used magnetoencephalography (MEG). Although both EEG and MEG signals reflect common neural currents, there has been controversy regarding the accuracy of EEG and MEG in locating brain sources. The performance of EEG and MEG is affected by many factors. EEG‐based source estimation does require an accurate conductivity profile of the head volume. In contrast, the electrical inhomogeneity of the magnetometer can be easily resolved, and an appropriate forward model can be obtained with a simple one‐compartment model. Meanwhile, MEG is mainly limited by its insensitivity to radial cortical sources. In addition, EEG reflects current sources in all directions. However, when the electric field gradient distribution is considered, the electric field gradient may be suppressed because of the low EEG conductivity, which makes the EEG signal susceptible to noise. In addition, the longitudinal source parameters can be more accurately estimated using EEG, while the transverse source parameters can be more accurately estimated using MEG. Thus, EEG and MEG are complementary in characterizing brain sources. The combination of EEG and MEG can improve localization accuracy.^43^ Luke Tait et al. evaluated the performance of six commonly used source reconstruction algorithms using human resting‐state MEG and generated a simplified cortical map of 230 regions from the Human Connectome Project's Human Cortical Multimodal Resolution, optimized to match the spatial resolution and MEG data level of the current generation MEG scanner. The results show that there is no “one‐size‐fits‐all” algorithm, and the appropriate algorithm selection was based on data and target analysis. For source‐level microstate analysis of EEG, specific evaluation of source reconstruction functions and parcellation atlas is necessary for diagnosis and clinical practice. Furthermore, combining EEG and MEG for source reconstruction may better maximize the potential for high temporal resolution to explore neuroanatomical markers of consciousness generation at the millisecond level.^44^

This study has several limitations to be considered when interpreting the findings. First, microstate segmentation may represent an oversimplified measurement of EEG dynamics. The “winner‐take‐all” labeling excludes the possibility of competing microstate classes during EEG segments and presumes discontinuous EEG evolution. We interpreted the results of our microstate analysis within the constraints of the GEV and clustering methods. Additionally, our approach yielded results that explained extensive variance in brain activity although these features seem clinically relevant. Furthermore, the interpretations of the eLORETA localization findings were limited by the relatively low spatial resolution of the inverse solution. Although this method has an inherent lack of precision, source localization facilitated speculation regarding the possible mechanisms underlying our microstate results. Finally, the small statistical sample size may not cover all causes of consciousness disorders and reduced the validity of the study.

## 5 CONCLUSION

This is the first study to conduct a microstate analysis of temporal dynamics at the source level in the EEG of patients with DOC. Our findings suggest that consciousness depends on complex dynamics within the brain and may originate from the anterior cortex. Our findings may contribute to the elucidation of specific and generalizable markers for consciousness during the generation and recovery processes as well as the neurophysiological mechanisms underlying human consciousness.

## AUTHOR CONTRIBUTIONS

CZ: visualization, writing‐original draft, methodology, formal analysis, and writing—review and editing. SH: data collection and formal analysis. XG and XC: data collection. LX: data collection and visualization. JH: resources, data curation, and writing—review and editing. YY: visualization, resources, data curation, and writing—review and editing. LB: resources and writing—review and editing.

## FUNDING INFORMATION

This work was supported by the National Natural Science Foundation of China (No. 81600919 and No. 81201980) and the Beijing Nova Program (Z181100006218050).

## CONFLICT OF INTEREST

The authors declare that the research was conducted in the absence of any commercial or financial relationships that could be construed as a potential conflict of interest.

## Supporting information


Figure S1

## Data Availability

Because of the privacy regulations of the hospital, the EEG and MRI data can only be accessible by contacting Dr. Yi Yang with research application.
